# On the Pathomorphological Pattern of the Efficiency of Photodynamic Therapy of Murine Melanoma B16 Using a New Photosensitizer Based on Chlorin e_6_ Conjugate with a Prostate-Specific Membrane Antigen

**DOI:** 10.3390/molecules27113445

**Published:** 2022-05-26

**Authors:** Olga B. Abramova, Grigory A. Demyashkin, Valentina V. Drozhzhina, Nina D. Yakovleva, Ekaterina A. Kozlovtseva, Tatiana P. Sivovolova, Petr V. Shegay, Sergey A. Ivanov, Andrey D. Kaprin

**Affiliations:** 1 A. Tsyb Medical Radiological Research Center—Branch of the National Medical Research Radiological Center of the Ministry of Health of the Russian Federation, 249036 Obninsk, Russia; dr.dga@mail.ru (G.A.D.); drozhzhina_1949@mail.ru (V.V.D.); yakovleva.40@mail.ru (N.D.Y.); beregovskayekaterina@gmail.com (E.A.K.); tchurikova.3144@yandex.ru (T.P.S.); oncourolog@gmail.com (S.A.I.); 2 National Medical Research Radiological Center of the Ministry of Health of the Russian Federation, 249036 Obninsk, Russia; dr.shegai@mail.ru (P.V.S.); kaprin@mail.ru (A.D.K.)

**Keywords:** photodynamic therapy, murine B16 melanoma, photosensitizer, pathomorphology, devitalization of tumor cells, immunohistochemistry, histology

## Abstract

Photodynamic therapy (PDT) is an effective treatment for a number of solid malignancies. In this work, the antitumor efficacy of photodynamic therapy for murine B16 melanoma with intravenous administration of a new photosensitizer (PS) based on the chlorin e_6_ conjugate with a prostate-specific membrane antigen (PSMA) was studied in vivo. We have previously published the data obtained in the first part of the study: the dynamics of PS accumulation in the tumor and surrounding tissues and the antitumor efficacy of the photodynamic therapy, which was evaluated by the regression parameters and morphological characteristics of the tumors—including by the complete regression of the tumors, the absolute growth rate of the tumors among the mice with continued tumor growth, and an increase in life expectancy compared to the control. The criterion for a complete cure was the absence of signs of tumor recurrence within 90 days after therapy. The conducted studies demonstrated the high efficiency of the new photosensitizer for the photodynamic therapy of B16 melanoma. This article presents a continuation of this work, including histological studies of the zones exposed to laser irradiation on the 21st day after treatment and an assessment of the therapeutic potential of photodynamic therapy for the destruction of tumor cells. Pathological studies in the zones of photodynamic exposure revealed that the effectiveness of the PDT depended on the PS dose and the laser irradiation parameters.

## 1. Introduction

The mechanism of photodynamic therapy (PDT) is based on the selective accumulation of photosensitizing drugs introduced into the body in cells with increased mitotic activity (tumor cells, endothelium of newly formed vessels, etc.). The uniqueness of the biological effect of PDT is explained by the induction of damage to biological structures via reactive oxygen species (ROS) and nitrogen oxides—natural regulators of cell proliferation, metabolism, and apoptosis. An important factor inducing a PDT-mediated response is damage to the cell membrane and tumor vessels. This selectivity of action determines the undisputed advantages of PDT—foremost, its ability to achieve the necessary therapeutic effect (radical destruction of the neoplasm) with minimal damage to the surrounding structures. Immediately after the PDT of tumors sensitized by photosensitizers, microcirculation disturbances, hemorrhages, and necrosis of most of the tumor tissue occur. To date, there are considered to be three main mechanisms for the antitumor effect of PDT: direct damage to tumor cells, the impairment of the blood supply to the tumor via damage to the tumor stroma vessels, and the elimination of malignant cells due to the cytotoxic effects of immune cells caused by the development of acute inflammation [1,2].

The progress in antitumor PDT is associated with the creation of effective photosensitizers (PSs) that provide a therapeutic effect with minimal damage to healthy tissues, high selectivity for accumulation in the tumor, rapid elimination from normal tissues, and the absence of any general toxic effects [3,4].

**Figure 1:**
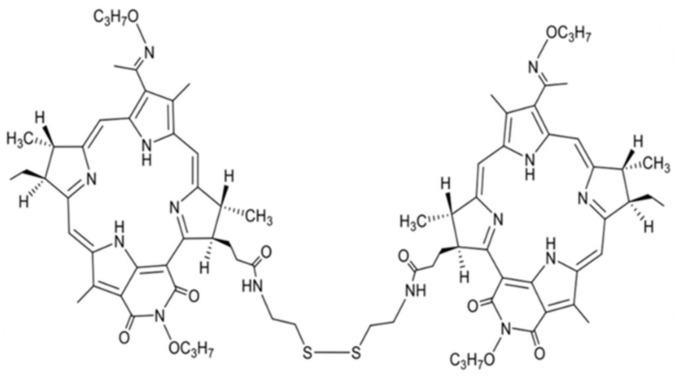
Structural formula of the photosensitizer conjugate of chlorin e_6_ with a PSMA-ligand.

In our work, we used a new photosensitizer—a conjugate of chlorin e_6_ with a prostate-specific membrane antigen (PSMA ligand; Figure 1) developed by Professor M.A. Grin, N.V. Suvorov, and A.F. Mironov [5]. The high efficiency of the PDT with this PS on prostate tumor cells (22Rv1) has been shown previously [6]. In our previously published work, the high photoinduced activity of this PS against B16 melanoma tumor cells was revealed [6]. The studies conducted so far have shown that laser exposure in the 45–60 mins after the intravenous administration of PS at a dose of 2.5–5.0 mg/kg with light exposure parameters E = 144 J/cm^2^, Ps = 0.48 W/cm^2^ causes 100% eradication of tumor nodules and the absence of their recurrence over 3 months of observation. Presumably, this result was achieved due to the high selectivity of PS accumulation in the tumor caused by PSMA ligand uptake by PSMA receptors in mouse melanoma cells [6,7,8,9].

The purpose of this study was to evaluate the antitumor efficacy of PDT with this photosensitizer against transplanted murine B16 melanoma according to the histological and immunohistochemical pattern observed in the area of laser radiation exposure on the 21st day after PDT.

## 2. Results

### 2.1. Photodynamic Therapy

The mice of the four experimental groups were intravenously injected with the PS at the rate of 2.5 or 5.0 mg/kg body weight. The light exposure parameters were: light energy density E = 105 or 144 J/cm^2^ and power density Ps = 0.25 or 0.48 W/cm^2^. The light spot diameter was 1.0 cm.

After the PDT, there was a pronounced soft tissue edema in the irradiation zone. The edema was accompanied by the hyperemia of the irradiation zone and surrounding soft tissues. Tissue alterations were observed, and a dense dark scab began to form 2–4 days after the treatment. Complete tumor regression was observed visually and by palpation in 100% of the animals in all experimental groups over 21 days of observation after treatment. In the control groups, the continued growth of the tumor nodules was observed (Table 1) [6].

### 2.2. Pathomorphology

For the morphological studies, the animals were euthanized by cervical dislocation on day 21 after the photodynamic exposure (day 30 after the tumor transplantation); the control animals were euthanized at the same time after the transplantation.

#### 2.2.1. Immunohistochemical Studies

The immunohistochemical studies were conducted in two experimental groups (group 1 and group 2) in which B16 melanoma was implanted under the skin of the thigh and subjected to PDT, in comparison to control groups that did not undergo PDT (control 1 and control 2).

##### Control Groups

**Figure 2:**
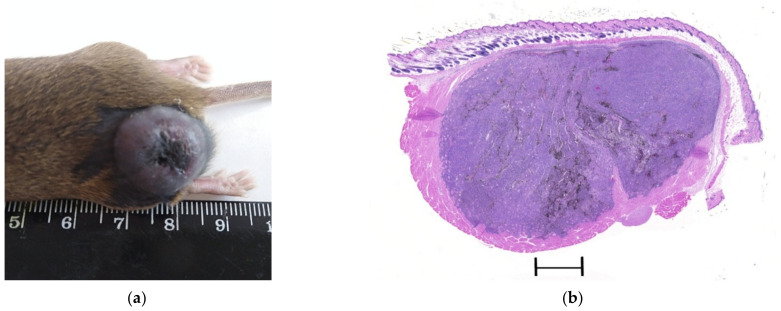

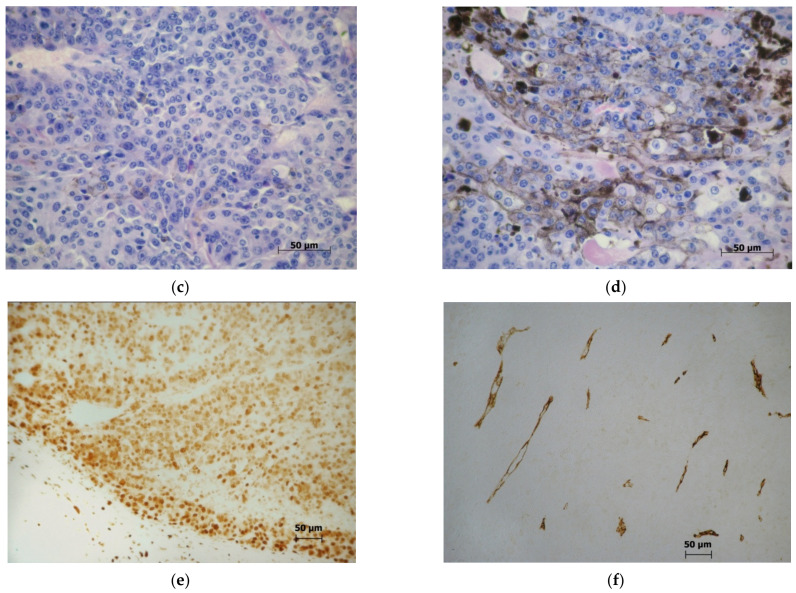
The morphology pattern of the B16 melanoma in the controls (control 1 and control 2) on the 30th day after the transplantation: (**a**) the macrophoto of the B16 melanoma on the 30th day after implantation under the skin of the thigh; (**b**) The histological pattern of the B16 melanoma on the 30th day after implantation. Staining with hematoxylin and eosin. Scan; (**c**) The histostructure of the B16 melanoma in the zone of solid growth. Staining with hematoxylin and eosin. ×40; (**d**) The melanin-containing cells in the tumor parenchyma. Staining with hematoxylin and eosin. ×40; (**e**) The PCNA-positive nuclei of the B16 melanoma in the peripheral zone of the tumor. Immunohistochemical staining for PCNA. ×20; (**f**) The vascular network in the solid zone of the B16 melanoma. Immunohistochemical staining for CD31; ×40).

Tumor-like mobile formations were determined on the outer surface of the left thigh of the animals (Figure 2a). The histological pattern of the melanoma on the 30th day after implantation in the mice of control group 1 is shown in Figure 2b. The areas of solid growth, with a well-developed vascular network, were represented by densely packed tumor cells (Figure 2c).

In the areas showing hypoxia and spontaneous necrosis scattered throughout the parenchyma, the individual melanoma cells were in a state of hydropic dystrophy. Melanin was present in the cytoplasm of the dystrophically altered cells, the structure of which varied from a dust-like granularity located along the periphery of the cell boundaries to the large granules densely filling the cytoplasm (Figure 2d). The immunohistochemical staining of the preparations revealed that the intensively proliferating PCNA cells were located in the peripheral zones of the tumors, concentrated around the vessels (Figure 2e). A developed vascular network was detected in the zones of solid growth when stained for CD31 (Figure 2f).

##### Experimental Groups (Group 1 and Group 2)

**Figure 3:**
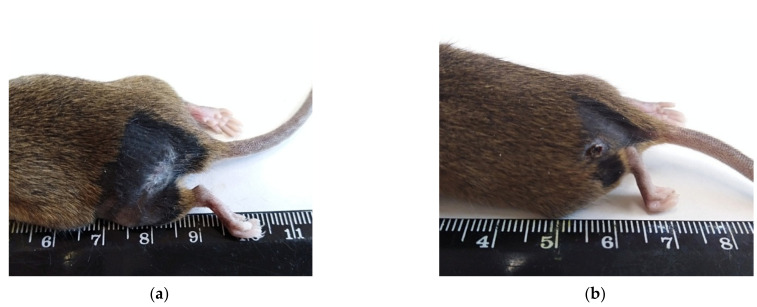
Healing of the skin in the area of the photodynamic exposure in the experimental groups on the 21st day after PDT: (**a**) Healing of the skin with scar formation. Macrophoto; (**b**) Healing of the skin with the formation of a scab. Macrophoto.

On the 21st day after the photodynamic exposure, the tumors were not detected in the area of the melanoma implantation macroscopically or by palpation. On the skin of the thigh in the PDT zone in the 1st and 2nd experimental groups, there were whitish, barely noticeable scars (Figure 3a) or brownish scabs (Figure 3b).

##### Experimental Group 1 (5.0 mg/kg; 144 J/cm^2^; 0.48 W/cm^2^)

**Figure 4:**
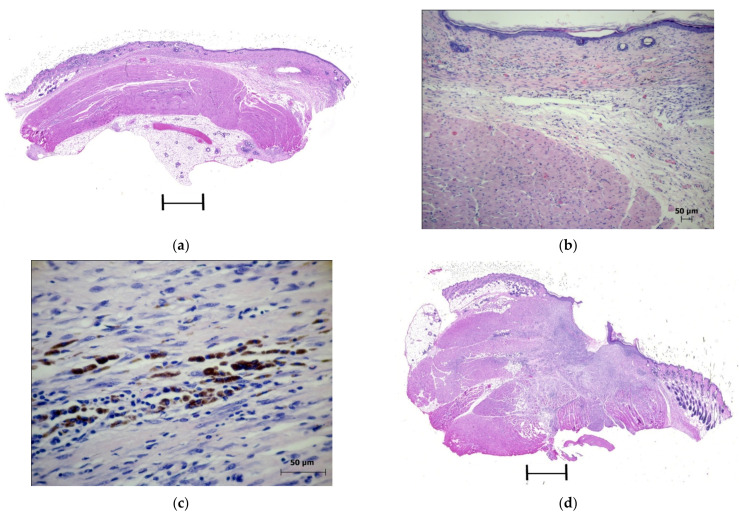

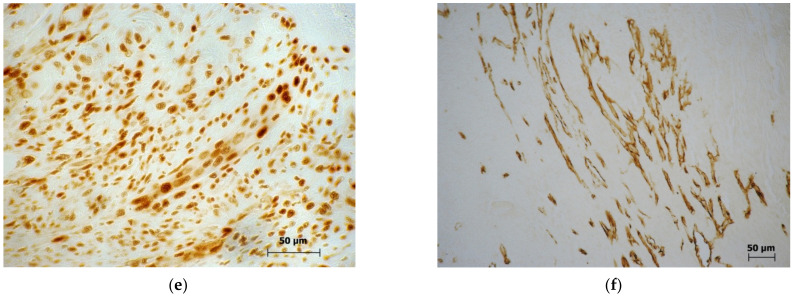
The laser exposure zone on the 21st day after the PDT. Experimental group 1: (**a**) The histological pattern of the zone of the photodynamic impact during scar formation. Staining with hematoxylin and eosin. Scan; (**b**) The epithelial regeneration and proliferation of the connective tissue in the area of the scar. Staining with hematoxylin and eosin. ×10; (**c**) Fragment. A group of melanin-containing macrophages at the site of an eliminated tumor in the zone of photodynamic exposure. Staining with hematoxylin and eosin. ×40; (**d**) The soft tissues of the thigh under the scab. Staining with hematoxylin and eosin. Scan; (**e**) The PCNA-positive nuclei of the regenerated epithelial and the connective tissue cells. Immunohistochemical staining for PCNA. ×40; (**f**) A capillary network in the muscle tissue of the thigh in the PDT zone. Immunohistochemical staining for CD31. ×40.

In the PDT zone, 58% of the mice had whitish, barely noticeable scars, while the rest of the animals had brownish scabs. The microscopic examination of the animals revealed the complete elimination of tumors. During the healing of the skin lesions in the PDT zone with the formation of a scar, epithelialization of the skin defect (Figure 4a) and a local proliferation of the connective tissue under the newly formed epidermis (Figure 4b) were observed.

At the site of the eliminated tumors, some clusters of macrophages containing melanin granules in the cytoplasm were usually observed (Figure 4c). During the healing of the area of the photodynamic exposure with the formation of a scab in the animals, the removal of necrotic tissues was associated with the formation of a zone of damage to the skin surface. The connective tissue located under the scab was infiltrated with round cell elements, among which no viable tumor cells were found (Figure 4d).

In the cells of the connective tissue that replaced the tumor nodules eliminated after the irradiation and the emerging epithelial regeneration in the basal layer of the epidermis, intensely stained PCNA-positive nuclei were detected. Immunohistochemical staining for CD31 in the PDT zone revealed that numerous vessels had formed in the area of the bottom of the skin defect (Figure 4e,f).

##### Experimental Group 2 (5.0 mg/kg; 105 J/cm^2^; 0.25 W/cm^2^)

**Figure 5:**
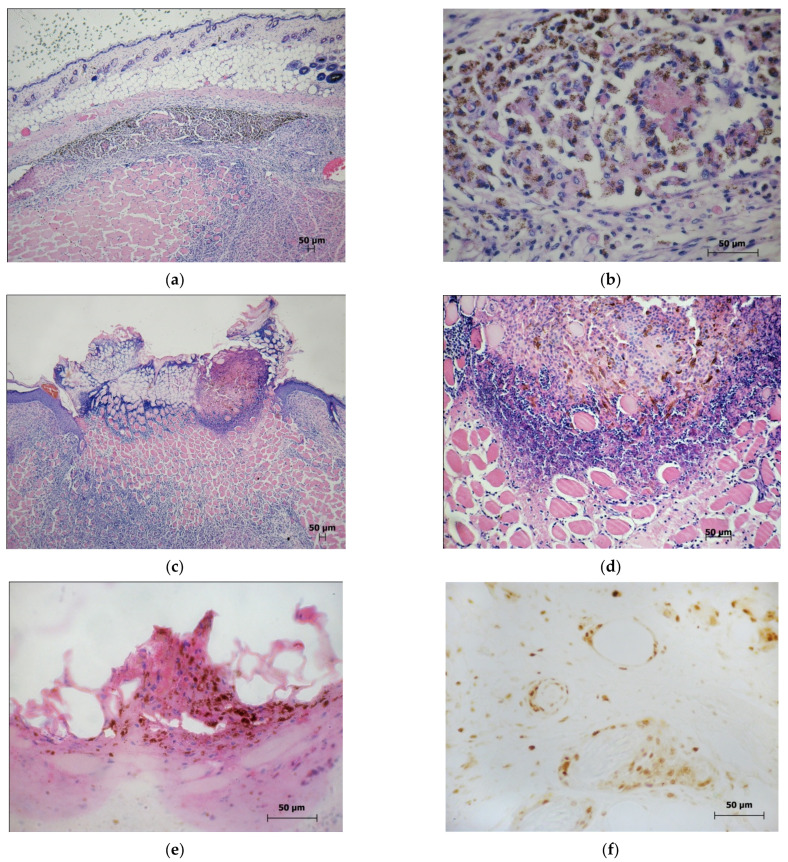
The zone of the laser exposure on the 21st day after the PDT. Experimental group 2: (**a**) Skin healing and melanin-containing macrophages in the zone of elimination of the tumor detritus. Staining with hematoxylin and eosin. ×5; (**b**) Fragment. The melanophages in the area of the melanoma photodestruction. Staining with hematoxylin and eosin. ×40; (**c**) The stroop in the PDT zone. Staining with hematoxylin and eosin. ×5; (**d**) Fragment. Staining with hematoxylin and eosin. ×20; (**e**) The eschar containing the tumor detritus. Staining with hematoxylin and eosin. ×40; (**f**) A PCNA-positive reaction of the nuclei of the endothelial cells and the connective tissue of the perineurium. ×40.

In the PDT zone, 67% of the animals had small dark-colored or light scabs and the rest of the mice had small light scars. In some animals with an epithelialized surface, the zone of the photodynamic exposure contained the remnants of the tumor detritus (Figure 5a), which were resorbed by the macrophages (Figure 5b). Other animals’ scabs were tightly soldered to the underlying tissues, consisting of dead skin fragments with epithelial appendages and necrotic muscle fibers, and containing the remnants of the tumor tissue (Figure 5c,d). The connective tissue located under the scab was infiltrated with round cell elements, among which no viable tumor cells were found (Figure 5e). In the nerve and the muscle fibers located in the damaged area, the response to the PCNA varied from weak to pronounced (Figure 5f).

Thus, with the irradiation parameters used in experimental group 1 (5.0 mg/kg; E = 144 J/cm^2^; Ps = 0.48 W/cm^2^), the effective elimination of the necrotic tumor nodules was observed, with the help of the melanophages migrating from the tumor elimination zone through the vessels of the lymphatic system. In experimental group 2, with lower parameters for the laser irradiation (5.0 mg/kg; E = 105 J/cm^2^; Ps = 0.25 W/cm^2^), the removal of the tumors that had undergone photodestruction occurred not only with the help of the melanophages—as in experimental group 1—but also by rejection as a part of a scab in some animals.

#### 2.2.2. Histological Studies

The histological studies were conducted in two experimental groups (group 3 and group 4) in which the B16 melanoma was implanted under the skin of the thigh and subjected to PDT; in comparison, the control groups did not undergo PDT (control 3 and control 4).

##### Control Groups

**Figure 6:**
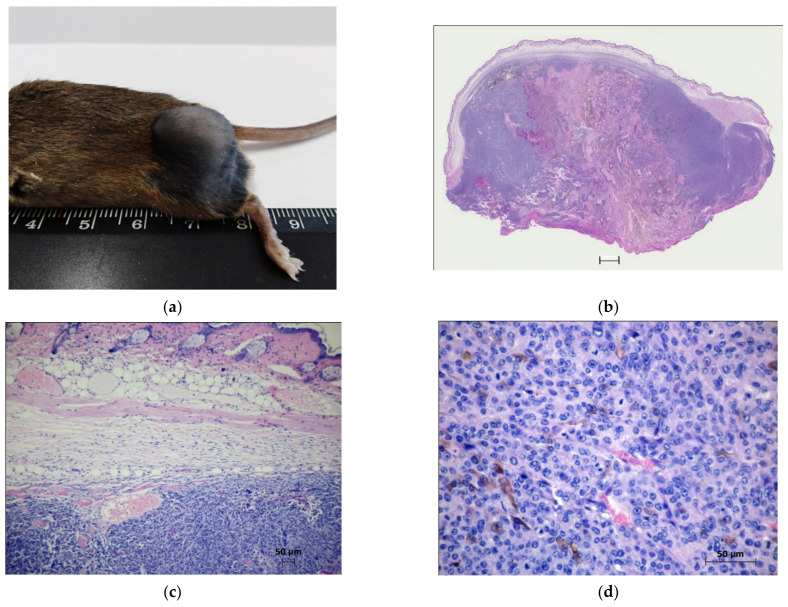

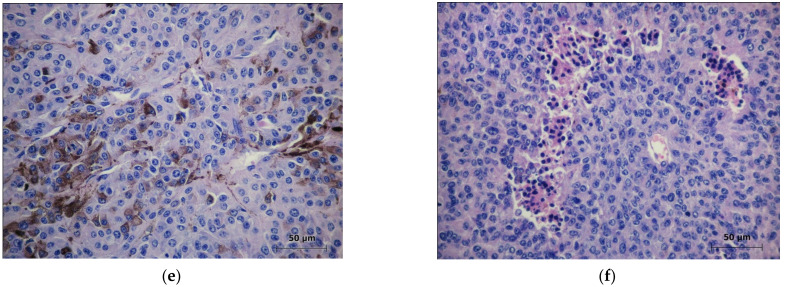
The morphology pattern of the murine B16 melanoma in the control groups (control 3 and control 4) on the 30th day after transplantation: (**a**) Macrophoto of the murine B16 melanoma on the 30th day after implantation under the skin of the thigh; (**b**) The morphological picture of the tumor on the 30th day after the implantation. Staining with hematoxylin and eosin. Scan; (**c**) A peritumoral zone of the melanoma 30 days after the implantation. Staining with hematoxylin and eosin. ×10; (**d**) A histostructure of the B16 melanoma in a zone of the solid growth. Staining with hematoxylin and eosin. ×40; (**e**) The melanocytes in the tumor parenchyma in the hypoxic zone. Staining with hematoxylin and eosin. ×40; (**f**) The micronecrosis in the parenchyma of the B16 melanoma. Staining with hematoxylin and eosin. ×40.

The isolated tumor nodules had a rounded shape (Figure 6a) and were dark gray when sectioned. The histological pattern of the melanoma 30 days after implantation is shown in Figure 6b. The tumors spread to the epidermis or subcutaneous adipose tissue. A significant part of the tumors was occupied by spontaneous necrosis, with the cuffs of the tumor cells surrounding the intact vessels. The zones of the solid growth were located in the peripheral parts of the tumors or were located in the form of narrow bands over superficially located necrosis (Figure 6c).

Mitotic cells were often observed (Figure 6d). Cases of apoptotic cell death were rare. Pigmented melanoma cells were found in the form of small clusters at the border with necrotic areas or in hypoxic zones, with a polygonal shape and long processes (Figure 6e). In the solid area of the parenchyma, dilated full-blooded vessels were present; microhemorrhages and micronecrosis were noted (Figure 6f).

The tumors of the mice from the control group with the introduction of the photosensitizer without irradiation, and from the group with laser exposure without the introduction of the PS did not differ from the tumors of the mice of the control group without any treatment.

##### The Experimental Group 3 and Group 4

In group 1 and group 2 on the 21st day after the photodynamic exposure in the area of the B16 melanoma implantation, tumors were also not detected macroscopically or by palpation. On the skin of the thigh in the PDT zone in the 3rd and 4th experimental groups, there were whitish, barely noticeable scars (Figure 3a) or brownish scabs (Figure 3b).

##### Experimental Group 3 (2.5 mg/kg; 144 J/cm^2^; 0.48 W/cm^2^)

**Figure 7:**
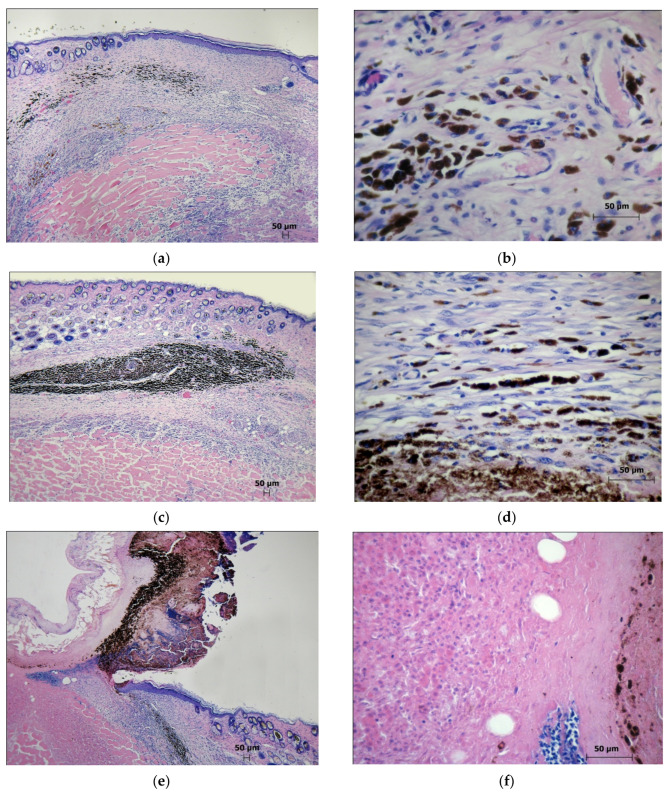
The zone of the laser exposure on the 21st day after the PDT. Experimental group 3: (**a**) Epithelialization of the zone of the photodynamic exposure and destruction of the muscle tissue. Staining with hematoxylin and eosin. ×5; (**b**) The melanin-containing macrophages at the site of an eliminated tumor in the zone of the photodynamic exposure. ×40; (**c**) The melanophages eliminating tumor detritus. Staining with hematoxylin and eosin. ×5; (**d**) An accumulation of the melanophages around the dilated lymphatic capillaries and in their lumen. Staining with hematoxylin and eosin. ×40; (**e**) A rejection of a part of the tumor eliminated by the macrophages in the composition of a scab. Fragment. Staining with hematoxylin and eosin. ×5; (**f**) Fragment. Histostructure of a necrotic tumor. Staining with hematoxylin and eosin. ×40.

On the skin of the thigh in the zone of the laser irradiation, in 83% of animals, there were whitish, barely noticeable scars; in the other 17%, there were dark-colored scabs. Microscopic examination of the PDT zone in animals with scar formation showed a complete epithelialization of the skin defects. Necrotized muscles were observed inside the connective tissue (Figure 7a) and scattered macrophages were detected (Figure 7b) in small groups or clusters within the disintegrated tumor (Figure 7c), with the cytoplasm containing melanin granules (melanophages).

Migration of the melanophages from the PDT zone along the connective tissue was noted; they were concentrated around the dilated lymphatic capillaries or were found in their lumen (Figure 7d). There was a rejection of a necrotic and melanophage-containing tumor in the composition of the scab (Figure 7e). At the same time, part of the necrotic melanoma remained in the PDT zone in the thickness of the dead muscle tissue (Figure 7f).

##### Experimental Group 4 (2.5 mg/kg; 105 J/cm^2^; 0.25 W/cm^2^)

**Figure 8:**
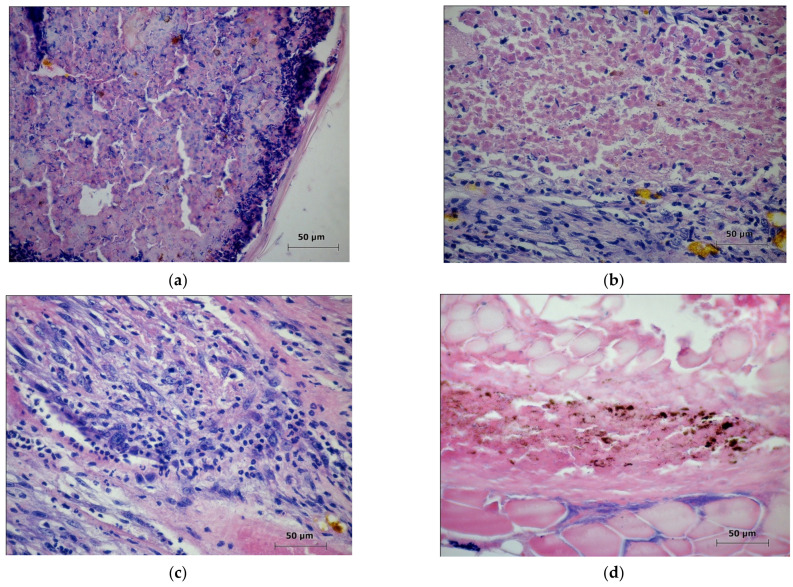

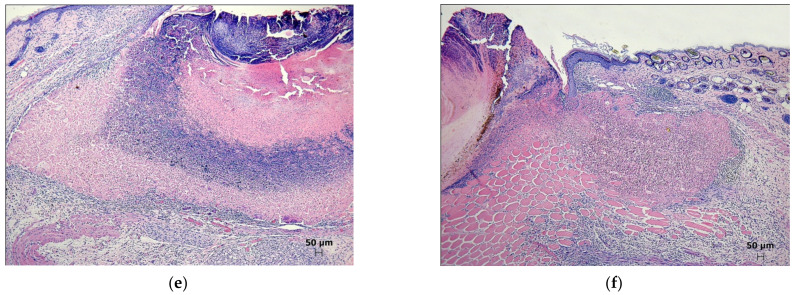
The zone of the laser exposure on the 21st day after the PDT. Experimental group 4: (**a**) Fragment. The histostructure of a necrotic tumor removed as a part of a detached scab. Staining with hematoxylin and eosin. ×40; (**b**) Fragment. A fragment of a necrotic tumor preserved in the PDT zone. Staining with hematoxylin and eosin. ×40; (**c**) Fragment. The microsection of a necrotic tumor containing viable B16 melanoma cells. Staining with hematoxylin and eosin. ×40; (**d**) The eschar in the PDT zone containing a necrotic tumor after the photodynamic exposure. Staining with hematoxylin and eosin. ×40; (**e**) Fragment. A part of a necrotic tumor limited by a leukocyte wall and located as a part of a developing scab in the PDT zone. Staining with hematoxylin and eosin. ×5; (**f**) Fragment. A part of an unremoved necrotic tumor located under the skin regenerated after the photodynamic exposure. Staining with hematoxylin and eosin. ×5.

In the 4th experimental group, 50% of the mice had small, dark-colored scabs in the PDT zone (Figure 8a) and 50% of the animals showed light scars. During the formation of the scars in one mouse, a fragment of a necrotic tumor was detected under the regenerated skin inside the connective tissue (Figure 8b). Additionally, one mouse had a microscab in a skin scar in the PDT zone, under which there was a small fragment of necrotic melanoma in which viable tumor cells were detected (Figure 8c). In two mice, the areas of the necrotic tumor were located under scars inside dead striated muscles. In one animal, in the PDT zone during the formation of a scab (Figure 8d), areas of tumor detritus located under scabs (Figure 8e,f) and partially surrounded by connective tissue were detected; in the remaining animals, the complete removal of the photodestroyed tumours was observed.

Thus, in the mice of the 3rd experimental group (2.5 mg/kg; E = 144 J/cm^2^; Ps = 0.48 W/cm^2^), the necrotic tumor containing the melanophages in the scab was rejected. Meanwhile, part of the necrotic melanoma remained in the PDT area inside the dead muscle tissue.

In the mice of the 4th experimental group (2.5 mg/kg; E = 105 J/cm^2^; Ps = 0.25 W/cm^2^), only 25% of them showed complete removal of the tumor nodules. In most mice where a scab or scar was present in the PDT area, remains (fragments) of the necrotic melanoma were detected. In some animals, areas of a necrotic tumor containing viable B16 melanoma cells were also detected.

## 3. Discussion

The results of the macro- and microscopic studies of the PDT zone on the 21st day after exposure to murine B16 melanoma indicate that the use of chlorin e_6_ conjugated with a PSMA-ligand as a photosensitizer in the PDT schemes used here had high antitumor activity.

In the mice of the 1st experimental group (5.0 mg/kg; E = 144 J/cm^2^; Ps = 0.48 W/cm^2^), the microscopic examination revealed the total elimination of tumors.

In the mice of the 2nd experimental group (5.0 mg/kg; E = 105 J/cm^2^; Ps = 0.25 W/cm^2^), microscopic examination also indicated the complete elimination of tumors. In some animals, removal of tumors that have undergone photodestruction occurs not only by the use of melanophages, but also by rejection as a part of a scab.

In the mice of the 3rd experimental group (2.5 mg/kg; E = 144 J/cm^2^; Ps = 0.48 W/cm^2^), necrotic tumors in scabs containing melanophages were rejected. At the same time, part of the necrotic melanoma remained in the PDT area inside the dead muscle tissue. Possibly, the remaining tumor fragments that were not removed during the study period would be further eliminated by the macrophage cells. The presence of viable B16 melanoma cells in the non-removed neoplasm fragments is not excluded and may cause the recurrence of tumor nodules.

In the mice of the 4th experimental group (2.5 mg/kg; E = 105 J/cm^2^; Ps = 0.25 W/cm^2^), only 25% showed the complete elimination of tumor nodules. In the majority of mice, in the presence of a scab or scar in the PDT area, remnants (fragments) of necrotic melanoma were detected. In some animals, areas of necrotic tumors containing viable B16 melanoma cells were also detected.

The reasons for the incomplete removal of tumors destroyed after photodestruction should apparently be associated with the topography of the subcutaneously located vessels, the distribution of the photosensitizer and its dose, the parameters of the laser irradiation, and the distribution of the photodynamic exposure energy in the biological tissue.

Thus, the comparison of the microscopic photographs of the results of the treatment of a malignant tumor by photodynamic therapy allows us to observe a clear picture of what happens in the irradiation zone on the 21st day after sessions with different exposure parameters.

With an increase in the dose of the photosensitizer and the energy density of the laser exposure (5.0 mg/kg; 144 J/cm^2^; 0.48 W/cm^2^) in the affected area, the effective elimination of necrotic tumor cells occurs. They are separated as parts of scabs or are excreted by melanophages migrating from the tumor elimination zone through the vessels of the lymphatic system. The number of remaining fragments of necrotic melanoma is minimal.

In the case of therapy with low doses (2.5 mg/kg; E = 105 J/cm^2^; Ps = 0.25 W/cm^2^), a significant number of remnants of necrotic tissue containing viable melanoma cells were observed in the affected area, which could cause recurrent tumor growth in the future.

## 4. Materials and Methods

### 4.1. Photosensitizer

The PS used in the study (Figure 1) was synthesized as described previously [5] and its properties were previously studied in prostate cancer cells [9]. The photosensitizer conjugate of chlorin e_6_ with a PSMA–ligand was kindly provided for research by Prof. M.A. Grin, Department of Chemistry and Technology of Biologically Active Compounds, Medicinal and Organic Chemistry, Institute of Fine Chemical Technologies, MIREA-Russian Technological University, Moscow.

### 4.2. Animals

The animal experiments were conducted in strict accordance with the Guidelines for the Care and Use of Laboratory Animals of the National Medical Research Radiological Centre of the Ministry of Health of the Russian Federation and in accordance with the rules and requirements of the European Convention ETS/STE No. 123 and international standard GLP (OECD Guide 1:1998). The animal experimental protocols used were approved by the Ethics Committee on Animal Experimentation of the National Medical Research Radiological Centre of the Ministry of Health of the Russian Federation (Permit Number: 1-D-00004).

Female F_1_ (CBA × C57BL/6j) mice (2–3 months old, 19–20 g body weight), were used in these studies. The animals were purchased from the Biomedical Technology Scientific Center of the Federal Biomedical Agency of Russia (Moscow). The animals were housed in T-3 and T-4 cages under natural light conditions with forced ventilation 16 times/h, at a room temperature of 18–20 °C and a relative humidity of 50–70%. The animals had free access to water and rodent PK-120-1 feed (Laboratorsnab Ltd., Moscow, Russia).

Antitumor activity was studied using the murine B16 melanoma model. The tumor strain was obtained from the tumor bank N.N. Blokhin National Medical Research Center of Oncology of the Ministry of Health of Russian Federation. Then, 2.5 × 10^6^ tumor cells in 0.15 mL of Hank’s solution (Pan-Eco, Moscow, Russia) were transplanted s/c into the area of the lateral surface of the left thigh in F1 (CBA × C57BL/6j) female mice.

On the 9th day after the neoplasia inoculation, when the tumor nodules reached a diameter of 0.4–0.6 cm, the animals were randomly divided into 4 experimental groups and 6 control groups (12 animals per group). As the series with the different treatment regimens were not performed simultaneously, each series had its own control. As a control, we studied tumor-bearing mice without any exposure, as well as a group with intravenous administration of PS at a dose of 5 mg/kg without laser exposure, and a group with laser irradiation without PS administration.

### 4.3. Photodynamic Therapy

On the same day, a session of photodynamic therapy was performed, as described previously [10]; the mice of the experimental groups received the PS in doses of 2.5 and 5.0 mg/kg into the caudal vein. The tumors were exposed to the laser irradiation using an Atkus-2 semiconductor device (Atkus, Russia; λ = 662 nm, diameter of the light spot = 1 cm). The following doses of laser irradiation were tested: E = 105 and 144 J/cm^2^; Ps = 0.25 and 0.48 W/cm^2^ (Table 2).

The antitumor efficacy of the PDT was assessed according to the criteria described earlier [6].

The animals were euthanized by cervical dislocation on day 21 after PDT (day 30 after tumor inoculation in the control groups). The development of neoplasia in the experimental and control groups was assessed morphometrically.

### 4.4. Histological and Immunohistochemical Studies

The isolated tissue of the tumor (control) or tissues of the area of the laser exposure in the form of plates oriented along the long axis and regional iliac lymph nodes were fixed for 24 h in Bouin’s fluid. Oriented tissue fragments were embedded in the Histomix paraffin medium at the HistoStar embedding station (Thermo Scientific, Cheshire, UK) after the standard histological wiring. Deparaffinized sections 5 μm thick, which were obtained on a Leica RM2235 microtome, were stained with the hematoxylin and eosin (BioVitrum, St.Petersburg, Russia) for the morphological studies.

Immunohistochemical studies were performed using polyclonal rabbit antibodies to the nuclear antigen of the proliferating cells—PCNA (Invitrogen, Rockford, Taiwan, PA5-27214; 1:100) and monoclonal rabbit antibodies to the endothelial marker—CD31 (Abcam, ab182981; 1:500). For the immunovisualization of the rabbit antibodies, secondary goat antibodies to the rabbit IgG conjugated with horseradish peroxidase (Abcam, ab205718; 1:1000) were used. Solutions for immunohistochemistry were prepared in phosphate-buffered saline.

Histological sections were examined under the AXIO Imager A1 microscope with microphotography on the Canon Power Shot A640 digital camera at 4 objective magnification levels: ×5, ×10, ×20, and ×40. Scanned images of the sections were obtained on a digital scanner “Nikon Super Coolscan 8000 ED” (scale line on scans = 1 mm).

The results obtained for the independent groups were statistically processed using the Statistica 6.0 software (StatSoft, Inc., Tulsa, OK, USA). The descriptive statistics parameters are presented as M ± SEM. The intergroup differences were evaluated using the Mann–Whitney U test. The differences were significant at *p* < 0.05.

## Figures and Tables

**Table 1:** Antitumor Efficacy of PDT (M ± SEM).

Group	Tumor Volume, cm^3^		
	Day 0 (the Day of the Therapy)	Day 10	Day 21
Control	0.04 ± 0.02	0.19 ± 0.04	0.94 ± 0.04
PS 2.5 mg/kg, Е = 105 J/cm^2^, Ps = 0.25 W/cm^2^	0.04 ± 0.01	—	—
PS 2.5 mg/kg, Е = 144 J/cm^2^, Ps = 0.48 W/cm^2^	0.03 ± 0.02	—	—
PS 5 mg/kg, Е = 105 J/cm^2^, Ps = 0.25 W/cm^2^	0.04 ± 0.02	—	—
PS 5 mg/kg, Е = 144 J/cm^2^, Ps = 0.48 W/cm^2^	0.04 ± 0.01	—	—

Note. “—” no tumor. Control (no treatment; PS without irradiation; irradiation without administration of the PS)—means for all 6 control groups. The volume of the tumor nodules in control groups did not statistically significantly differ.

**Table 2:** Experiment Design.

Group	PS Dose, mg/kg	E, J/cm^2^	Ps, W/cm^2^	Evaluation Method
1 group-PDT	5.0	144	0.48	immunohistochemistry
1 control	0	0	0	immunohistochemistry
2 group-PDT	5.0	105	0.25	immunohistochemistry
2 control	0	0	0	immunohistochemistry
3 group-PDT	2.5	144	0.48	histology
3 control	0	0	0	histology
4 group-PDT	2.5	105	0.25	histology
4 control	0	0	0	histology
5 control	2.5	0	0	histology
6 control	0	144	0.48	histology

## Data Availability

Not applicable.

## Sample Availability

Not available.
